# Why Engage Communities in Biomedical Research?

**DOI:** 10.1111/bioe.70155

**Published:** 2026-07-16

**Authors:** Robert Steel

**Affiliations:** ^1^ Clinical Center Department of Bioethics NIH Bethesda Maryland USA

**Keywords:** community engagement, philosophy, research ethics

## Abstract

While community engagement in biomedical research is widely lauded, and many reasons to engage communities have been adduced, it is unclear whether all these reasons are equally important, and, if not, which are most important. Here, I argue that, outside of rare circumstances, community engagement should not be understood as seeking permission from communities. Rather, it should be understood as adding value to research. I also argue that while community engagement may add value in a variety of ways—including making research more respectful, more empowering, and more likely to successfully translate into health gains—it is the health gains that should be its primary focus.

## 1 Introduction

It is increasingly common, and encouraged, for biomedical researchers to engage the communities with which they plan to run their trials through mechanisms such as, for example, empaneling community advisory boards. But there is more consensus *that* community engagement is important than there is on *why*. For instance, Seema Shah, Frank Miller, and Holly Fernandez Lynch, summarizing the literature, identify a full fifteen distinct potential goals which have been put forward for community engagement in biomedical research, all of which appear *prima facie* reasonable, and which lack any hierarchical ordering or further arrangement [1, p. 887].^1^ While it's plausible that community engagement simultaneously serves multiple legitimate purposes, one difficulty with such a multitude of goals is that it is unlikely that they will always be in harmony and unclear what to do when they conflict.

This paper is particularly concerned with how to understand and relate two widely recognized, high‐level goals of engagement that subsume many of its more particular ambitions: on the one hand, the goal of realizing respectful and empowering relationships, and, on the other, that of making research more efficient in promoting health. It asks: does respect impose constraints on how researchers treat communities—for example, requiring certain forms of engagement—that simply take precedence over considerations of efficiency, or can they be traded off against each other? And, if they can be traded off, how should we think about those tradeoffs? While existing requirements and guidelines, as well as the language used to discuss community engagement, sometimes suggest implicit answers to these questions—answers which are not always aligned—they have been subject to little sustained research‐ethical analysis.

On one dramatic potential answer, engagement should aim at securing a form of permission or authorization from the community that is analogous to the informed consent of an individual, which would indeed imply that the right to respectful treatment which underlies it is one that cannot traded off against mere efficiency. But I argue that in most cases community engagement should not be conceived of as serving such a consent‐seeking function. This is because when research occurs against a background of legitimate state institutions, it already receives the relevant form of community consent just by virtue of complying with applicable law and regulation. By contrast, in cases under thoroughly illegitimate background institutions, a consent‐seeking community engagement process may be necessary; even there, however, I argue that it can be legitimate to proceed regardless. I suggest that which cases are which be assessed on analogy to making treatment decisions for an incapacitated patient.

So, in most cases, realizing respectful and empowering relationships through community engagement should instead be thought of as a way of contributing additional value to the research—including, perhaps, an intrinsic ethical value directly associated with those relationships. Hopefully the values of empowerment, respect, and efficient health promotion generally align, such that respectful treatment also best promotes health. But this cannot be assumed and, while existing data on the impact of engagement are inadequate to determine exactly when these values work together and when they work at cross‐purposes, there are theoretical reasons to suspect they must sometimes come apart. So we must confront questions like the following: if more extensive engagement processes were routinized in a way that led to both genuinely more empowering relationships with host communities, yet also a decrease in the quantity of studies logistically feasible to conduct and an associated reduction in the pace of research, would that be an overall good or bad thing? To assess such tradeoffs, I first describe a case study illustrating how community engagement may promote all of these values, and then argue on its basis that, if there are forced choices, then the values of empowerment and respect will justify at most small setbacks to health promotion.

## 2 What Is Community Engagement, and What Should It Be?

One NIH group has defined community engagement as “a process of inclusive participation that supports mutual respect of values, strategies, and actions for authentic partnership of people affiliated with or self‐identified by geographic proximity, special interest, or similar situations to address issues affecting the well‐being of the community of focus” [2, p. 1383–5]. But nothing in the discussion to follow hinges on the specifics of this definition as opposed to any other. Similarly, while I will speak throughout of “community engagement,” there are other, related terms which are sometimes used interchangeably and sometimes distinctly, and this paper does not aim to taxonomize or distinguish them. The very general phenomenon being discussed here is just the addition of some form of contact, at minimum informative and possibly accompanied by authority or control, that links researchers and communities and which is distinct from those interactions that always occur just due to study participation itself.

I also take a general approach to the research being considered. Work on community engagement often discusses it in the context of a specific kind of research—for example, field trials, controlled human infection studies, xenotransplantation trials, community‐based participatory research, public health research—however, despite that specificity, the discussions often either depend on or advance more general ethical claims about communities and researchers. This work aims to engage with those more general claims.

Across research types, methods for engaging communities include, non‐exhaustively, empaneling community advisory boards, holding public forums, maintaining bulletin boards, question boxes, or public‐facing websites, presenting at religious and civic organizations, speaking on the radio, meeting with professional societies, and incorporating members of the public into the grant review processes. Multiple methods of engagement may be used at the same time, and meaningful engagement may require the use of multiple methods.

Community engagement in research has long been a US regulatory requirement for emergency research where the collection of individual consent would be impracticable [3], and its use is frequently required or recommend for internation research [4] and research that involves aboriginal communities [5, 6, 7]. Specific sets of good participatory practice (GPP) guidelines have been developed to guide engagement in HIV prevention research [8], tuberculosis drug [9] and vaccine [10] research, COVID‐19 research [11], and research for emerging pathogens [12]. The Council for International Organizations of Medical Sciences (CIOMS) recommends community engagement generally, though it adds that it is “especially important when the research involves minorities or marginalized groups” [13, p. 25]. The Patient‐Centered Outcomes Research Institute (PCORI) is notable for requiring a community engagement component for all funded projects [14].

While support for community engagement is broad it is also, in at least one respect, thin: both guidance and requirements typically avoid giving explicit decision‐making authority over research to communities. For instance, in interpreting the aforementioned US regulatory requirements for waivers of consent for emergency research, the FDA maintains that the goal of community consultation is to inform communities and solicit input into the research and that this input ought to be considered; notably, there is no requirement that it be complied with [15, p. 24–34]. And when discussing appropriate the appropriate roles of researchers and communities within the engagement process, CIOMS maintains that researchers must be sensitive to local culture and values, but also that communities must not be allowed to make stipulations that compromise the scientific value of studies; when it comes to the possibility of irresolvable disputes between the two, the guidelines simply state that “it is important to specify in advance who will have the final say” [13, p. 26].

Is this reticence defensible? Perhaps not. Consider how Ngui's chapter on community engagement in the *Roberts Academic Medicine Handbook* sorts community engagement activities into three grades of intensity and the language it uses to describe each [16]. The lowest grade, transactional engagement, “is largely a consultative form of engagement, a form of charity engagement… one‐way… [and] top‐down… Community acts as a passive recipient…. Decisions are usually paternalistic, made by academics or agencies ‘for’ the community but not ‘with’ the community.” By contrast, the intermediate grade, transitional engagement, involves “moving further” through “seeking cooperative work… two‐way consultation, and some collaboration,” but nonetheless still “falls short by not fully engaging communities in power sharing and control of resources or the process of leadership and decision‐making found in transformational engagement.” The highest grade, transformational engagement, is “marked by high levels of collaborative and participatory decision‐making, leadership, and empowerment among partners…. Interrogates power structures and imbalances…” and is “characterized by more shared leadership relationships.” These descriptions are accompanied and illustrated by a figure in which the modes of engagement are arranged left‐to‐right in an upward‐reaching arc that leads from “less shared leadership” to “most shared leadership,” a progression which is also emphasized by a large arrow [16, p. 350–1].

This presentation does not explicitly state any particular normative requirement; nonetheless, given the language and framing, it is natural to wonder if an ideal is nonetheless being strongly suggested. Relatedly, it is also natural to wonder whether, for example, the FDA specifying that only outreach and consultation (rather than collaboration and shared leadership) are required for emergency research means that it falls short of the suggested ideal, and perhaps even lapses into “top‐down paternalism.”

Alternatively, consider the following from David R. Buchanan, writing on community‐based participatory research (CBPR) in the *Oxford Handbook of Public Health Ethics*: “just as individual research participants can never be treated merely as a means to an end, communities should also have the collective right to decide if they want to participate in any proposed research… CBPR is a significant if imperfect (in its current form) solution to this critical ethical challenge” [17, p. 350]. While this chapter's topic is specifically CBPR, note that it positions CBPR as answering an ethical challenge which is fully general to research, namely, ensuring that “communities should have the right to decide whether participation is consistent with their collective goals and interests” [17, p. 346]. But a right to make a decision cannot be secured just by outreach and nonbinding consultation.

Stepping back, while community engagement is both broadly encouraged and frequently praised, the terms of the praise often imply that it would be appropriate for this to be less a matter of encouragement and more one of requirement, and that requirements should specifically be for a robust form of shared authority.^2^

## 3 Should Community Engagement Aim at Community Consent?

Buchanan, above, draws an analogy between individuals' and communities' rights to decide whether they participate in research. But is it in fact normatively necessary to secure permission from a community before conducting research in that community in the same way that it is normatively necessary to secure permission from an individual before conducting research on that individual? There are two potential routes by which one might argue that it is.

Here is the first: research can impose risks not just on participants, but on community members as well. For instance, controlled human infection studies (CHIs) deliberately infect participants with diseases for scientific purposes. While efforts are made to minimize the risk of the disease under study leaking out into the community, this risk often cannot be fully eliminated. Participants are, through the informed consent process, made aware of the risks that infection poses to them and have the right to refuse participation. But under current US regulatory standards, there are no specific limits on the risks that can be imposed on nonparticipant and no requirement to obtain their consent. Yet, as Holly Fernandez Lynch points out, this difference in levels of protection provided is hard to square with the apparent moral equality between participants and bystanders, and one natural way to resolve this tension would be to extend the protections currently given to participants to bystanders as well [19].^3^

One could do so simply by requiring that bystander risks never be imposed, which would *a fortiori* mean they were never imposed without consent. A “near‐zero risk for nonconsenting bystanders” standard was articulated and applied by a commissioned report on the ethics of proposed Zika CHI trials, with the authors writing that “[b]ecause… third parties generally cannot know about, protect themselves from, or consent to risks, risks to them are only reasonable if they can be reduced to near‐zero” [20, p. 19]. But a distinct strategy would be to allow bystander risks, but require that they be consented to. Indeed, the idea that community consent might be an important solution to nonparticipant risks is natural enough to at least receive mention in the context of xenotransplantation, where one of the focuses of ethical attention is on the bystander risks associated with potentially enabling interspecies spread of pathogens [21, p. 63–4; 22, p. 459–61]. Similarly, in the context of studying genetically‐modified insect vectors, Macer writes: “obtaining the consent of any person potentially affected by the release of transgenic insects, is important for the ethical conduct of research trials, whether or not national guidelines require this, or even exist” [23, p. 653–4]. Community engagement could serve as the mechanism by which such consent from all potentially affected individuals would be solicited.

But if the point of community engagement were to extend the very same consent‐related protections against risk that are currently afforded to participants to nonparticipants as well, this would suggest that engagement should be practiced very broadly, not just in, for example, CHI or xenotransplantation studies. While those categories do impose clear medical risks to nonparticipants, essentially any clinical trial will have secondary effects that include risks, burdens, and inconveniences to trial nonparticipants, even if only as minor as increased traffic and difficulty parking around the study site, and in the context of participant consent even minimal risks and burdens require soliciting consent and respecting refusal whenever it is feasible to do so.^4^ This rationale also implies that the relevant standard for obtaining community permission should be unanimous consent. For competent adults enrolling in a trial, *each* individual must consent for *themselves* to enroll in a trial where there will be nontrivial research risks or burdens. If *any* refuse to accept those risks or burdens, they cannot be enrolled; and it doesn't matter whether that person refusing means the trial cannot go forward. Similarly, it suggests every individual should retain a right to withdraw at any time without penalty, even if that too requires the research stop.

The argument for requiring community engagement on the grounds that it is an essential mechanism for vindicating nonparticipants' moral right to refuse risk impositions has the virtue of a straightforward motivation and clear implications, but it also has the unattractive feature that it makes research impossible, as nearly all research has third‐party effects on large groups from which obtaining unanimous consent would be unrealistic. But is there any more principled reason to reject it?

Fortunately, yes: the starting principle that there are no morally legitimate impositions of risk and/or burdens on individuals without consent is implausible on examination; whatever moral principle underlies and explains the right of potential participants unilaterally decline research participation, it is not just that everyone has the fully general right to reject risks.^5^ To the contrary, ordinary life involves the routine nonconsensual imposition of small risks and burdens. For example, when I move into an empty apartment, I impose the burdens of my noisy presence on my hapless downstairs neighbors. Whenever I drive, I impose a tiny risk of a fatal accident on each pedestrian I pass. But neither neighbors nor pedestrians have any right to be asked before I do those things. This is not just true as a matter of law, but rather seems desirable and necessary. We live in a world where our actions have many different effects on many different people, and it is unclear how it could even in principle be possible to give every individual who might be affected by a decision a unilateral veto over it. Suppose that leaving my apartment to drive very marginally increases the risk of death to a pedestrian, but also that my other option, of remaining in my apartment, very marginally increases the chance of my floor collapsing and killing my downstairs neighbor. I must do one or the other, so it cannot be that both parties have a right to unilaterally veto the course that risks harm to them. What if they both said no?

But while it is a nonstarter to treat any community member who might be negatively affected as having a right that requires researchers to secure their consent *as individuals*, perhaps these observations are consistent with, and even support, the idea that researchers must obtain the collective consent of the community *as a community*. In terms of the previous example, while it does not make sense to think of any particular pedestrian as having an individual right to forbid me from driving past, or any particular apartment‐dweller to tell me to stop standing above them, it does make sense to think that my community as a whole should be able to determine how people are allowed to drive and how sturdy their floors are. This is the second route to arguing that community engagement could serve a permission‐seeking function.

Understanding the community itself as the appropriate target for engagement rather than the individuals who make it up is congruent with typical language, as in the various GPP guidelines and both textbook chapters cited above. And because positing a community‐level right better handles the reality that social life ineliminably involves nonconsensual risk‐imposition, it is more plausible than the individualistic argument.^6^ Neuhaus, writing specifically in defense of a community authorization requirement for conducting field trials which release genetically modified animals, gives eloquent voice to this kind of motivation: “Requiring the community authorization process for field trials is a way of respecting persons affected by the decision, and it gives authority to communities to decide what happens in their backyards, to their pets, and on their children's playgrounds” [24, p. 31].^7^

There is something clearly correct in the idea that communities should have the right to determine the rules under which they operate. However, such considerations do not support a requirement to obtain communal permission for the conduct of research specifically through a community engagement process. This is because appropriate self‐determination can be, and already is, accommodated in other ways. Specifically: when research is conducted in democratic states, the background mechanisms of democratic politics already give communities the opportunity to organize and lobby politically for their interests, including their interests surrounding medical research, and to give the decisions they reach in that manner the binding force of law and regulation.

In democratic states, the rules and regulations governing research, as well as the bureaucrats and officers who interpret and enforce them, ultimately answer to the people. In the view of most, this conveys normative authority. So, while communities do have a right to permit and forbid activities of common concern, such as research in their back yards and on their playgrounds, they are already appropriately exercising that right through their duly constituted democratic governments, and researchers are already in the business of obtaining permission from those governments just by virtue of following applicable local law and regulation.^8^

McCoy, Dellgren, and Emanuel raise a related challenge in the distinct context of public engagement for the purposes of health priority‐setting, for example, determining the content of national insurance schemes: they allege that proponents sometimes write as if democratic legitimacy were an on‐off property, and, for that property to be on, the public must be directly engaged through processes such as citizen's juries [27]. But this seems to presuppose that only direct democracies are legitimate, which is surprising given that electoral democracies are by far the most common form. The vast majority of state action doesn't occur after direct consultation, and could not, in virtue of how cumbersome and expensive it would be to try to delegate every discretionary decision currently made by a politician or bureaucrat to a direct participatory method.^9^ It is significantly more plausible to treat public engagement not as necessary for a decision to be democratically legitimate at all, but instead as a way of enhancing democratic legitimacy, and to focus its use on specifically those cases where the background mechanisms of representative democracy function most poorly and therefore stand in greatest need of improvement.

Here, relatedly, I am claiming that following the law and regulation of a legitimate state is already sufficient for acting with the permission of the community. This is nonetheless consistent with the idea that community engagement can enhance the democratic quality of that permission along some graded dimension and that enhancing its quality may add ethical value, especially in those cases where background institutions function poorly.

The distinction between seeking permission *tout court* and enhancing the quality of permission in a way that adds ethical value may seem slight, but it carries significant implications. When it comes to securing permission from individuals for their participation in research through the informed consent process, this is, in most cases, a hard constraint. Under regulation and prevailing ethical thought, it is never permissible to say that “no one would ever consent to being in this research study (say, because it is very painful and permanently disabling), yet it is *very* socially valuable, therefore we do this to people without their consent.” While it is possible to waive consent for sharply delimited kinds of research, this is not an allowable reason—because, where required, informed consent is understood as a constraint that is prior to and cannot be traded off against social value. If community engagement were to seek permission in an analogous sense, obtaining it would also be a constraint that could not be traded off against other values. That is why it is necessary to first make the arguments in this section, that what is at issue in community engagement is not securing permission *tout court*, and only after that does it make sense to go on, as I later do, to think about the value that enhancing the quality of permission might add to research and how to trade it off against other values when they conflict.

## 4 How Enfranchising Does Government Need to Be to Convey Community Consent?

But, before going on to consider value tradeoffs, first entertain an obvious objection. While it is true that in theory democratic governance enables all to participate on equal terms in collective decision‐making, some states are not democratic. And even those that are, and which embrace equality as a *de jure* standard, nonetheless suffer from material and social inequalities that lead to *de facto* uneven distributions of power. One might thus question whether the democratic processes just mentioned can be relied on to effectively represent, and thus offer permission on behalf of, politically marginalized communities. For example, for a research trial which aims to enroll gay men or sex workers, it may be that in the relevant jurisdiction these groups are highly stigmatized, and it may therefore seem willfully obtuse to claim that consistency with national law and regulation, or some national health official's decision to approve a trial, constitutes a normatively relevant form of community consent on behalf of those groups. This is particularly so given that the standard history of research abuses includes entries in which public health authorities in democratic countries approved the mistreatment of politically powerless communities.

In response, I first acknowledge that there are situations in which some states cannot speak on behalf of some internal communities, and in such situations something other than the legitimating force of democracy would need to be invoked to explain the appropriate governance of medical research with those communities. This seems obviously true in extreme cases, for example, research in states that do not even formally represent themselves as being democratic, let alone *de facto* enfranchise their many internal communities. The interesting questions, then, are how serious failures of either *de jure* or *de facto* representation need to be before the above arguments cease to hold and what to do when they are.^10^

One reason to take the failures to need to be serious is in order to avoid blanket skepticism about legitimate state authority. The regulation of medical research is not the only arena in which states make decisions governing common life, and, presumably, if the existence of marginalized communities were to compromise the state's ability to convey normatively necessary forms of community consent to research, it would also compromise the state's ability to convey normatively necessary forms of community consent with respect to for example zoning, renter's rights, fire risk, taxation, food safety, traffic, noise, air quality, indecency, and everything else. Essentially every policy states adopt affects marginalized communities, and if those affected communities' consent to such policies were both normatively necessary and absent under conditions of marginalization, then, given that real‐world governance is inarguably disappointing and every state contains marginalized communities, the implication would be a form of *a posteriori* anarchism, the view that while legitimate state authority is coherent in theory, no real‐world governments exercise it. Instead, we should be willing to allow that even flawed processes can often produce normatively authoritative consent on behalf of involved communities, provided those flaws are not too obviously disqualifying.

One could resist this line of thought by maintaining that we should endorse a form of research exceptionalism on which community consent to research is different in kind to community consent to any other public activity, or, relatedly, just by rejecting the relevance of broader theoretical considerations to this specific case. This objector might maintain that, in the concrete case we are considering, requiring communities be engaged and give permission before research is conducted would empower and protect marginalized groups without incurring any comparably morally important costs or drawbacks, and so we should just do that: rather than counting groups as adequately represented by prevailing law and regulation unless *de jure* or *de facto* failures of effective representation are very severe, we should do so whenever they exist at all, or perhaps even potentially exist.

Yet this proposal does incur costs and drawbacks. One practical problem is that community engagement requires time and resources, so more broadly construing the cases where it's required potentially increases research burdens. But, bracketing that, another problem is that it's not clear that authority over research with a community *should* reside with just that community.

Consider, as an example, recent research which not only enrolled gay men but also had the potential to effect gay men as a class: a French trial of DoxyPEP as an intervention for the prevention of bacterial STIs in gay men [30]. Of course, every individual enrolled in that trial gave their informed consent to participate; in that sense, the authority over research with those men already resided with those men. But, beyond the participants, the trials also had implications for French gay men as a whole: both the prevalence of STIs and potential rise in antibiotic resistance stood to impact not just participants themselves but also their sexual networks, and, in addition to those more directly medical effects, they also had the potential to further shape cultural norms around for example, condomless sex in ways welcome or unwelcome. These effects make it reasonable to think that the broader community should be able to influence the design of such trials and, more strongly, perhaps to be able to decide whether this was a research question that should be investigated at all.

But in the same way that research with some gay individuals can affect other gay individuals and gay men as a class, it can also affect non‐gay‐men and non‐gay‐classes. In this case, successful research in gay men has predictably led to trials in other demographics. Perhaps more centrally, the potential successful control of bacterial STIs—or, unfortunately, an increase in antibacterial resistance in STIs—has implications for all sexually active people in France; as decades of public health work emphasizes, sexual networks are overlapping and the health of key populations is interlinked with the health of the population as a whole.^11^ And that is just in consideration of the direct health effects. Additionally, stakeholders throughout the health system will have their own practical and commercial interests implicated whenever a new intervention is under consideration.

So, while the earlier reasoning about broader community effects is compelling, it also lacks a natural stopping point. Insofar as gay men as a class can have a legitimate interest in whether and how research proceeds with some subset of individual gay men, society as a whole can also have the very same kind of legitimate interest in whether and how research proceeds with gay men. Hence the motivation for generalizing beyond the *specific gay men* enrolled in a trial–who, again, give their own informed consent entirely separate from whether any broader community is or is not engaged–to gay men generally, appears to equally well support generalizing beyond the class of gay men.

This is not to say that there was a serious conflict among the interests of different segments of French society over DoxyPEP trials, especially given that DoxyPEP was already being used off‐label and rigorous research could only improve practice. In this case, it seems that most stakeholders would have approved regardless of how they were selected. Rather, the point is that it appears artificial to restrict the relevant community to just French gay men. Ideally, decisions about research design and conduct would be responsive to a maximal synthesis of affected interests, rather than strictly prioritizing any group. Indeed, this idea does not contradict and if anything is instead reflected in existing guidance on community engagement, which tends to take a maximal scope in considering affected populations, for example treating stakeholders as including anyone who is affected by the conduct or non‐conduct of research.^12^ Taken literally, the group of all potentially affected individuals plausibly includes nearly every living and future person. Yet it is not clear how to engage nearly every living or future person.

This point here is to bring out the implausibility of the suggestion that there is a neat, research‐exceptional answer for who has authority over medical research, one that is different from and clearer than answers for who has authority over for example zoning, taxation, or clean air. Medical research also involves interconnected and sometimes conflicting interests across society, and, when it comes to thinking through how to balance them, simple answers (e.g., give exclusive control to discrete communities, identified somehow) are not any more plausible than they are elsewhere.

Against this backdrop of normative uncertainty, the currently existing entities that currently engage the broadest scope of people using procedures that we are most confident confer legitimacy are democratic states. Democratic states allow a broad spectrum of interests to be brought to bear on issues of common concern through the mechanisms of electoral democracy and produce results which can speak on behalf of the enormous group of subject‐stakeholders whose claims were expressed and aggregated. And while, as initially acknowledged, there are situations in which the operation of a given state will be so transparently indifferent or abusive to a discrete internal community that we should not take that state to be offering any form of normatively relevant permission on that community's behalf, the fact that democratic states supply the best current answer we have to this question implies that we should construe these exceptions narrowly rather than broadly.

Note, importantly, that this argument is fully consistent with allowing that one of the things a state can use its authority to do is create a regulatory requirement that researchers, in some cases, engage communities directly—as noted, this is already the case in the United States with respect to obtaining waivers of consent for emergency research. This argument is also consistent with the claim that doing so would be an excellent idea, particularly when it comes to types of research where *de facto* democratic oversight is weak or problematic. Rather, the claim here is just that we should be relatively forgiving when we assess the level of democratic quality necessary for states to have the authority to make the decision whether or not to adopt such a policy in the first place, and, for states that do meet that bar, unless and until they actually impose those requirements we should take conformance to their general law and regulation as conveying any normatively necessary form of community permission.

## 5 Research When Local Law and Regulation Do Not Convey Community Consent

Now suppose that we are considering research within a state whose political procedures do not meet even a realistically plausible standard with respect to enfranchisement of a research‐relevant group. There are various attempts to operationalize liberal democracy and rank countries around the world; suffice it to say that no scale ranks every country as any kind of democracy, let alone a full one. So, imagine that researchers are for example, considering a study in an authoritarian state that would enroll a disfavored minority group which is in violent conflict with central authorities. In at least some cases, it will be clear that a government cannot offer normatively significant permission on behalf of at least some internal populations.

Arguably, research in contexts with difficult politics is required not just in order to discharge humanitarian duties but duties of justice as well, and a discussion which restricted its scope to research conducted in adequately‐functioning democratic states would therefore be incomplete [32]. Ask, then: for researchers to ethically conduct research under such conditions, must they develop and deploy legitimacy‐conferring community engagement processes to obtain permission before they proceed?

I suggest the answer is “sometimes.” To determine when, these cases can be approached through an analogy between individuals and states. A well‐functioning democratic state is relevantly like a capacitated patient. Researchers can ascertain and respect its wishes, which are presumptively authoritative in matters regarding its treatment. States which are not at all democratic, or in which democratic procedures are unable to adequately enfranchise a particular vulnerable community under study, are instead like incapacitated patients. More strongly, such states are like an incapacitated *unrepresented* patient, as there is also no independent surrogate authorized to speak on their behalf. And designing a community engagement process which can offer (or withhold) normatively significant community consent is relevantly analogous to attempting to restore capacity to an unrepresented patient so that they can then make their own treatment decisions.

In the case of patients, this is frequently both possible and appropriate. When an unrepresented patient's capacity could be easily restored–for example, through waiting a few hours–and the treatment decisions they face are significant and highly value‐sensitive, yet not particularly time‐sensitive, it is obviously appropriate to restore capacity before proceeding. However, there are also cases where it is instead appropriate to proceed directly with treatment under a best interest standard, for instance when the prospects of restoring capacity are distant or uncertain, the patient's condition will deteriorate without treatment, and/or the choices being made are only minimally value sensitive. When unconscious victims of car accidents are brought to the emergency room unaccompanied by friends or family, they are medically stabilized regardless of the fact that neither they nor any surrogate is available to consent. Many cases are intermediate between such extremes and deciding whether to attempt to restore capacity or to proceed under a best interest standard requires a weighing of factors [33].

I submit that, in the case of research with what we could call “unrepresented communities,” a weighing of factors will often support proceeding without first attempting to analogically restore capacity through designing and implementing a normatively authoritative community engagement process. Consider, as an example, research into the health effects of coating roofing surfaces with relatively inexpensive substances that can increase solar reflectance and lead to cooler indoor temperatures. This intervention may reduce the negative health effects of heat stress, which is especially pressing in low‐resource settings challenged by increasing global temperatures. However, this question needs to be studied, and, ideally, studied in a wide range of contexts in which different roofing materials are used (for instance, one study in Burkina Faso is applying liquid applied membrane coatings to roofs common there, for example mud brick, tin, and thatch) [34]. It is unlikely that any comprehensive world‐view held by majorities around the world views heat stress as something that ought to be left as it is. And, to the extent that interventions are delayed or foregone, morbidity and mortality from heat will likely increase. Conducting research into cool roofs even when community permission cannot be secured therefore seems relevantly analogous to appropriately treating an unrepresented patient, as delaying will lead to harms and the intervention itself is not particularly value sensitive.

Because this approach balances factors, it will sometimes come out the other way. Research into the empowerment of socially stigmatized groups may be highly socially controversial and in some cases it may be that community consent would be necessary. If so, then not just any engagement process would be adequate—it would need to be one that secures a normatively authoritative form of collective permission, a standard which is conceptually obscure and likely logistically intensive. It may follow that it is generally infeasible to proceed with research that would require community consent in areas where there is no government capable of offering it.

The standard should not be that researchers must receive permission whenever it is so much as conceivable that some stakeholder somewhere could have a values‐based objection. Human psychology is variable, countries are heterogenous, and some individual or another will always be available to object. Rather, what is relevant is the degree of uncertainty regarding what, were there to be adequate democratic processes in play, they would decide. When the value‐sensitivity of a research intervention is significant and this rises to the level that researchers are genuinely unsure whether appropriate democratic processes, if they were to exist, would find their work acceptable, that factor that counts strongly against proceeding. I doubt this of much research along the lines of the cool roofs intervention described above, but it is ultimately a matter of study and judgment in particular cases.

## 6 Kinds of Value Added Through Community Engagement

If community engagement aimed to secure community consent in a manner relevantly analogous to individual consent, that would supply a relatively clear answer to how to balance its various goals: the goals related to ensuring the validity of that consent would take precedence, just as securing the validity of informed consent takes precedence over other concerns when enrolling individuals in research. But I have argued that we should not conceive of community engagement this way, and thus a different answer is required.

The most straightforward alternative, which I will spend the rest of the paper exploring, is that community engagement adds several different kinds of value to research, and balancing its various goals therefore requires thinking about and comparing them. This section sets up that discussion by offering a brief overview of a case that shows these different kinds of value: the history of the development and dissemination of PrEP for prevention of HIV, starting first with the failures of large planned PrEP trials in Cambodia and Cameroon in the early 2000s and then moving on to some contemporary difficulties with uptake [35, 36].^13^

At the time, the drug Tenofovir Disoproxil Fumarate (TDF) was already in use for treating people living with HIV, but trials were planned to test whether regular TDF, taken by people without HIV, would prevent HIV acquisition. Safety data did not yet exist for TDF in people without HIV, and the trials were planned as placebo‐controlled combined safety and efficacy trials that would enroll female sex workers in Cambodia and women at high risk of acquiring HIV across sites in West Africa, prominently including Cameroon; these populations had high incidence of HIV, increasing the trials' power to detect a prophylactic effect.

Both trials did carry out community engagement, but in each case, the existence and value of this engagement was not fully visible to those outside the trial. In Cameroon, funders designed the trial on the basis of extensive formative research with women who were likely to enroll and their immediate associates; however, in order to preserve the integrity of the social science they separated the responsibility for this formative work from the responsibility of running the trial, and, to uphold participant privacy, they refused to identify participants in that earlier formative work. The team was thus in a difficult position, with a lack of transparently available information fueling speculation about cover‐ups. In Cambodia, by contrast, the engagement was not limited to formative research and involved many public meetings; however, the public demands being made on the team were difficult to accommodate, with a particular sticking point being the conflict between a demand for post‐trial insurance and NIH's inability to obligate resources post‐trial. While the team did pursue rather than ignore the issue, that pursuit was not public‐facing and left participants in the public consultation feeling stonewalled by repetitive and uninformative meetings.

Additionally, in both cases research teams came into conflict with organized activist groups. In Cambodia, the Women's Network for Unity, a sex worker's union, pressed demands on behalf of their membership. In Cameroon, two members of the local NGO Réseau Ethique Droit et Santé (Network for Ethics, Laws, and Health) had become concerned about the trial, and, in cooperation with ACT Up Paris, played a crucial role in drawing media attention through arranging coverage through a sensationalistic French news program.

Some concerns about the trials touched on real issues in what were then emerging and unclear areas in research ethics, such as post‐trial access and care for participants who screened out as already living with HIV or who seroconverted on study; indeed, some of these were identified in an earlier expert research ethics consultation. Other concerns, however, were not substantive: in Cameroon, after feeling shut out of productive dialogue, activists sought to command attention by putting forward anecdotal claims whose provenance they had not adequately verified and sometimes repeated false charges (e.g., that the informed consent forms were only available in English). In both cases, critical attention spiraled in a feverish media environment, including unsourced reporting and posting on internet message boards where lurid rumors proliferated, such as that the study product contained HIV, or that researchers were hiring men living with HIV to have sex with participants to facilitate testing the study drugs' protective effect. In both cases, the teams lacked the media training necessary to respond effectively. As a result of the public controversy, the trials were cancelled by the Cambodian and Cameroonian authorities.

While those trials did not succeed, oral PrEP was nonetheless still investigated and later found to be highly effective–when taken consistently, oral PrEP prevents transmission of HIV more effectively than condoms. It was approved in the United States in 2012 and is now a grade A preventative that insurers are legally obligated to cover with minimal or no cost sharing. In Sub‐Saharan Africa (SSA), while coverage varies, some ministries of health have made oral PrEP broadly available, especially to high‐risk groups (e.g., couples in mixed‐serostatus relationships).

Unfortunately, despite its efficacy and increasing availability, oral PrEP faces barriers to uptake among some of the populations that need it most. The highest burden area for HIV disease is SSA, making it a priority for preventative interventions, and in SSA, incidence is higher among women than men. However, trials have found low rates of oral PrEP initiation, adherence, and persistence in women in SSA, relating to a number of barriers, perhaps the most commonly discussed being stigma [37, 38, 39, 40].

One strategy to address these barriers is to develop contraceptive multipurpose prevention technologies (cMPTs), for instance, pills or vaginal rings that combine both PrEP and hormonal birth control. These products may be more discrete and acceptable, and there is evidence of women's interest in cMPTs over single‐purpose HIV prophylaxis [41, 42, 43]. But the marquee success story in this space is the development of long‐acting injectable PrEP, which requires infrequent administration in the privacy of a clinic and also offers advantages in terms of feasibility and acceptability. In 2024, roughly a decade after the development of oral PrEP, the widely celebrated PURPOSE 1 trial of a twice‐yearly injectable formulation reported 100% efficacy in preventing HIV in a large cohort of adolescent girls and young women in South Africa and Uganda (zero infections out of 2134 participants in the active arm) [44].

The story of the development of PrEP illustrates two risks trials face: first, controversy leading to political pushback and cancellation, and second, developing products that, while medically effective, translate into smaller than expected health gains due to unforeseen barriers. Community engagement does not fully eliminate but can reduce these risks. This was recognized with respect to the first risk by UNAIDS and AVAC, which led to the development of the GPP Guidelines in response to the failure of the Cambodian and Cameroonian trials. With respect to the second risk, I speculate that more effective integration of community perspectives into research planning could have led to earlier and faster recognition of the barriers for oral PrEP in crucial populations and quicker pursuit of strategies for addressing them. It is notable that in the decade since oral PrEP was introduced, PrEP‐based cMPTs were never developed; it remains the case that condoms are the only approved product for prevention of both HIV and pregnancy.

Effectively managing these risks both makes trials more likely to succeed and makes the successes more valuable when they occur. Provided the cost isn't disproportionate to the benefit, that improves the efficiency of research at promoting health. The value of that gain in efficiency just is the value of the additional health that is being gained. Since these are two different ways of naming the same thing, I will hereafter speak interchangeable of efficiency and health value.

In addition to enhancing health value, community engagement also holds out the promise of realizing respectful and empowering relations with host communities. Looking to PrEP again, we can also see these values at stake. Engaging the women the trial sought enroll and their communities has the potential to empower them, giving them greater control over their lives and futures, and possibly also yielding psychological and material benefits in esteem and social position. This may have been a rare opportunity, as the women in question were otherwise subject to extreme clusters of overlapping disadvantage: in Cambodia at the time of the proposed PrEP trial, one study of sex workers in Phenom Penh found that within the last year 75 percent reported having been beaten by police and 49 percent reported having been gang‐raped by police [35, p. 9]. Furthermore, the empowerment of the specific individuals engaged could have positive spillover effects insofar as they may go on to promote further change.

I categorize all tangible positive effects of engagement under the idea of empowerment. This includes material and psychological benefits: both acquiring valuable skills or connections and experiencing improved life satisfaction would be empowerment benefits on this way of speaking. By contrast, I categorize intangible benefits under the idea of respect. Perhaps it is important for its own sake that individuals be treated respectfully, especially those who are otherwise severely mistreated. This value might also be subject to its own version of a spillover effect; it may be that by treating community representatives respectfully one thereby also treats the entire target population with respect. Perhaps the fact, even if entirely unknown to me, that someone has respectfully engaged with members of my community before proceeding with research may make me objectively better off; or, even if we don't think of it that way, it may simply make the world a better place that this happened, and that betterness may be irrespective of whether it ever affects or leads to anything else.

## 7 Do Respect and Efficiency Always Go Together?

Engagement can produce efficiency‐, empowerment‐, and respect‐related value, and in the PrEP trials engagement could plausibly have advanced all three in tandem: the right strategy may have been able to both prevent trial failure while also demonstrating respect toward and empowering participants and communities. But will these values always go together, and how should we think about their potentially coming apart?

In exploring this question, it will be helpful to start by considering a view recently articulated by Bridget Pratt. Pratt argues that co‐constructing knowledge is a moral necessity in public health research, and, hence, that public health research should involve not just informing or consulting with communities, but a form of power‐sharing that involves shared decision‐making in all phases of research projects [45]. There are three reasons it is useful to consider this view. First, Pratt's argument does not depend on the mistakes we criticized earlier: she does not claim that communities have a right to consultation nor does she argue from an analogy between individual and community consent to participation. Instead, she proceeds from the claim that co‐constructing knowledge adds important ethical value to research. Second, while her argument is applied to public health research, the values that she draws on plausibly extend to research more broadly. And third, the co‐construction she calls for requires a particularly robust form of engagement, and it is with robust forms of engagement that the potential for tradeoffs is most vivid.

Pratt argues that co‐constructing knowledge best advances two core moral aims of public health research, namely (1) addressing the health needs of the disadvantaged and (2) building relations of equality by supporting self‐determination and self‐development. Co‐construction helps to address the health needs of the disadvantaged by drawing on a broader range of social knowledge in order to solve the complex problems which impede their health and wellbeing, and it facilitates the self‐determination necessary for equality by including in decision‐making those significantly affected by it. Members of society are generally affected by decisions in public health research, as people's ability to meet their health needs depends on what preventative interventions are available to them, which is in turn downstream of the conduct of health research, and those significantly affected would include people living in the research setting or who are part of the groups under study. Additionally, inclusion in research decision‐making supports a “right to research,” developing beneficiaries' capacity to inquire, analyze, and communicate, thereby furthering their self‐development and helping to create relations of equality with researchers.

The first core aim of addressing the health needs of the disadvantaged, and the potential for engagement to allow researchers to draw on otherwise inaccessible knowledge in doing so, map onto the benefits for efficient health promotion discussed previously. The second aim, of building relations of equality through supporting self‐determination and self‐development, also maps onto both respect and empowerment. But what of the relation between them?

If these forms of value were always aligned, we would not need to consider this question. However, there is reason to suspect that the tangible benefits of empowerment must at some point come to conflict with efficiency (the case of the intangible benefits of respect is more complicated, and its discussion is deferred to the next section).

Why must empowerment and efficiency come apart? Consider that, in order to be empowering, engagement must be meaningful, and this may require material support for the individuals being engaged—for instance, to minimize barriers to participation and power differentials, it may be important to train community representatives in health and clinical trial literacy, to offer financial support to facilitate travel and time away from work, and so on. But while such measures may reduce the barriers to participation and power differentials, they do not eliminate them. A pamphlet describing site randomization does not equalize the knowledge of a research grantee and a community representative. One could, at progressively increasing cost, do more: rather than providing educational materials, one could conduct an intensive course; rather than an intensive course, one could offer an accredited degree‐conferring program. Rather than preserving financial neutrality through stipends for meals and travel, one could offer positive financial assistance adequate to securing freedom from near‐term want; beyond economic security for an engaged individual, one could supply economic security to dependents and associates. The problem is that the cost and delay would eventually threaten the underlying research project; we can imagine fanciful budgets where the funds set aside for these measures represent most of the expenditures. Such a budget could make sense, but only if the trial's goal were in the first instance to empower community representatives, with generating scientific knowledge that improves human health secondary to that.

Returning to Pratt, Pratt allows that widespread adoption of a co‐construction approach will require some significant changes to how trials are conducted: a shift in funding away from short‐term project‐based cycles, possibly to a system where funding also covers support for engagement during planning phases prior to full grant submission; training a next generation of researchers in new skillsets; and building trust, relationships, and capacity in way that will likely require longer timeframes and perhaps more resources [45, p. 812]. It is plausible these measures would generally promote empowerment. They may also promote efficiency through some pathways, for example, as Pratt argues, through genuine collaboration improving the uptake of information from consultations [45, p. 811]. At the same time, in allowing that research may also become more resource intensive, and slower, it is also clear how this relatively involved form of engagement may harm efficiency. What is the overall balance of these competing effects? This is ultimately an empirical question that can't be resolved through speculation.

Ideally, we would be able to consult a robust evidence base. Unfortunately, as both optimists and pessimists about engagement acknowledge, this is currently lacking [46, sect. 7]. A recent review of reviews finds instead that the impact of engagement activities is inconsistently reported, and that those reports that exist are of weak methodological quality, with impacts mostly being assessed qualitatively and predominantly through the retrospective self‐report of involved parties: it concludes that the data is sufficiently poor that it is difficult to understand the impact of increased research investment into engagement [47, p.]. So while there are strong theoretical reasons to believe that there must eventually come a point at which empowerment and efficiency must come apart, it is unclear where that is. It is also unclear for any specific model—such as robust co‐construction of the form Pratt advocates—whether it is advancing both or trading one off against the other.

## 8 Tradeoffs Between Respect and Efficiency

Whether and how engagement trades off different values is empirically unclear, and we cannot say for a particular approach that it is making a particular tradeoff. Nonetheless, it is possible to think hypothetically about how such tradeoffs should be made, even if we cannot say exactly where they are occurring. We can do that by thinking about how these different values relate to one another. To that end, return to the earlier example of failed PrEP trials in Cambodia and Cameroon.

First, start by just considering the total health value at stake in the conduct of these trials. What was lost by their failure? For participants, the fact that they did not receive PrEP surely led to many more HIV acquisitions, and for society the delay in its development and rollout likely contributed to significant additional morbidity and mortality. PrEP is a groundbreaking product, and in 2023 over 3.5 million people received oral PrEP at least once [48], with the subsequent development of long‐acting injectable PrEP having even greater promise. This entire branch of research may have moved faster if early trials of oral PrEP had succeeded rather than failed, and if we had been able to deploy these interventions even somewhat more quickly that would have created staggering health value.

That there was a great deal of health value at stake doesn't on its own mean that the efficiency‐related value of community engagement would have been large. After all, the trials did carry out some engagement yet failed anyway; perhaps the conditions were such that it was impossible to succeed regardless of how they tried. If so, there would have been no efficiency‐related value to be had. However, because of the particularly political nature of the trial failures, including the key role played by widespread mistrust and misinformation, it is at least plausible that there was an engagement strategy that could have enabled them to go forward. And the value of such an approach averting their failure would have been equivalent to the entire health value of the trials, which, as noted, could have been staggering.

Now consider the empowerment‐associated value at stake in these trials. As mentioned, it is possible that engagement could empower individuals in ways that offer them lasting benefits, and even potentially cause spillovers; perhaps the subsequent efforts of the empowered might lead to durable change in the social position of this severely marginalized community. If so, the value of empowerment could be considerable. Yet it is reasonable to be skeptical, as it is difficult to effectively intervene on social orders. Absent compelling evidence, which as earlier noted does not exist, a more plausible default is to assume that empowerment is, on average, mostly experienced by those who are directly engaged.

So considered, there seems to be much less of this value at stake than health value. Engaging a person on a community advisory board is time‐limited; preventing a person from acquiring HIV is a significant health benefit that may be lifelong. The numbers of people affected are also radically different. A community advisory board may have relatively few members; town halls will draw a bigger crowd, but will still involve many, many fewer people than the number who stand to benefit from the development of a novel medical intervention like oral PrEP. There is also a natural tension between the magnitude of the empowerment benefit and the number of people benefited: the more intensive the co‐construction, the fewer people it is feasible to include. So, while it is hard to directly compare empowerment to good health, the fact that so many more people's health is affected by research than will ever be engaged through a community engagement process, especially any intensive one, and that these effects are durable rather than transient, signals that there is considerably more health than empowerment value at stake.

Finally, consider the respect‐related value at stake in these trials. When it comes to the intangible value of respect, it is unclear how to estimate it, or whether to think of it in terms of quantities at all. Instead, I offer a different analysis. For that, it will again be helpful to begin by dividing the cases where research is conducted with a population in a jurisdiction where background institutions convey community permission from those where it is not.

If we proceed under the assumption that following existing law and regulation was already adequate for securing community permission from the women participating in these trials, then respect also seems already to have been satisfied. Pratt emphasizes the moral principle that decision‐making should give a say to those substantially affected, and the potential value contributed by engagement processes that directly involve those affected by public‐health research in that decision‐making; but recall the earlier argument that individuals already do have a say in those decisions, even without that separate direct involvement, by virtue of living under adequate background political institutions. It is not apparently disrespectful to engage in a course of action over which relevant parties have had a fair say, and where the result of that process was to permit it. Even if it is sometimes more respectful to check again, it is difficult to imagine that whatever value there is in doing so would be sufficient to justify trading away any meaningful health gains.

By contrast, things are different if we proceed under the assumption that their background institutions functioned poorly enough that they could not convey community permission. I have already suggested that in this kind of case we can make productive use of an analogy to decision‐making for an unrepresented patient, and I now return to that method here. Consider the following case:

Mr. X. is an elderly man who presents alone at an emergency room and then loses capacity. He is admitted to the hospital, and stabilized, but does not regain capacity. The doctors also incidentally discover a suspicious mass, though this is believed to be unrelated to Mr. X's primary complaint and his ongoing lack of capacity. Mr. X. did not name a surrogate, and the hospital's initial attempts to identify and contact an appropriate decision‐maker reveal that he lives alone, is not in contact with his family, and keeps to himself. The initial efforts to identify and contact his family have been unsuccessful.

Mr. X.'s narrow health interests support an immediate biopsy: if the mass is cancerous, delaying diagnosis and treatment risks progression and worsening prognosis. Yet it is difficult to say how much risk escalates with delay. It is also difficult to predict when or whether either attempts to restore Mr. X's capacity will succeed or a suitable surrogate will be identified.

As described, it is reasonable to continue attempting to restore capacity and/or contact a surrogate before addressing the mass. This enables consent to the biopsy, and if it shows the mass to be cancerous, then that will also require a series of further value‐sensitive decisions about treatment for which it would be greatly preferable that they be made by Mr. X or his surrogate. Waiting involves a setback to Mr. X's narrow health interests for the sake ensuring that Mr. X (or his surrogate) make the decisions, but the health risk is unclear and it is an open possibility that Mr. X's capacity could be restored or a surrogate contacted at any moment.

With time, the situation could change. It could become clear that: Mr. X will not regain capacity; that there is no appropriate surrogate; and/or the unidentified mass is a worsening and imminent health threat. It would require clinical and ethical judgment to say when, but at some point the health risks of waiting will have sufficiently waxed, and the prospect of ensuring that Mr. X or his surrogate makes the decisions will have sufficiently waned, such that it will become ethically appropriate to initiate treatment.

We should *not* describe that as the point where the threat to Mr. X's health interests comes to outweigh the value of respecting him. That description wrongly implies that declining to intervene until Mr. X dies would be the most respectful way to treat him, and it is only because of other values, outside of, opposed to, and eventually overwhelming respect that we must eventually initiate treatment. But this isn't true: permanently declining to intervene would not be the most respectful thing to do. Rather, what we should say is that respect for Mr. X may not be exhausted by but does at least *include* consideration of his health. Since it is not exhausted by consideration of his health, respect can require some setbacks to his narrow health interests. But because it does include that consideration, there is a limit to how significant those setbacks can be before allowing them instead becomes clearly disrespectful.

I submit that this is also what we should say about the potential for tradeoffs between the intangible value of respect for communities and the health‐related value of trials. Possibly, the former could require at least some setbacks to the latter. Perhaps engagement procedures which ensure this form of respect could require slightly longer timelines, slightly more funding, or result in slightly less research throughput. However, because respect incorporates caring about substantive outcomes, this value cannot require the adoption of procedures which result in large sacrifices to efficiency. It would show lesser, rather than greater, respect for West African and Cambodian women burdened by extremely high HIV incidence to engage them in a particular way if that were to substantially slow progress in the development of effective prophylaxis.

To summarize, the health‐related value of trials will often be significantly larger than their empowerment‐related value. And when it comes to the intangible value of respect: it is arguably satisfied at baseline when research is conducted under adequate background conditions, and, when that is not the case, the degree to which it can conflict with efficiency is limited by the fact that respect itself includes concern for health.

Working out what these claims imply for particular engagement procedures would require significant further work; it would not only need to address details of the approach and context, but also likely need to be informed by rigorous empirical data. So this argument is not directly about particular procedures, but instead about how to understand the justificatory structure of the underlying values. It is fully consistent with this argument that extremely resource‐intensive, highly empowering and respectful engagement methods could be the all‐things‐considered best approach. The implication is just that, if so, it would have to be because those approaches were also optimal (or nearly so) at efficiently promoting health.

## 9 Conclusion

Community engagement should not generally be thought of as aimed at securing community consent: engaging with communities to secure their consent is redundant in cases where political institutions function well, and, in those where they don't, only necessary in a subset. Instead, community engagement should be thought of as adding value to research. Those values include health value as well as empowerment and respect‐related value. These may often run in parallel, such that the same procedures simultaneously promote all, but it is implausible that this is always true. Given weak existing evidence we cannot identify where tradeoffs between them are occurring, but we can still draw some limited conclusions: specifically, we can conclude that in cases where tradeoffs arise, the relatively greater magnitude of value associated with efficient health promotion as opposed to empowerment, and the fact that the value of respect itself incorporates considerations of efficiency, support the prioritization of efficient health promotion with at most minor deviations.

## Conflicts of Interest

The author declares no conflicts of interest.

## Data Availability

The author has nothing to report.

## Endnotes

1 Those goals are: (1) protecting communities in research, (2) minimizing the potential for exploitation of participants and communities, (3) informing risk/benefit assessment, (4) understanding and respecting cultural differences and the local context, (5) showing respect for participants and other stakeholders, (6) being transparent and diminishing the spread of inaccurate information, (7) building appropriate levels of trust/increasing legitimacy of research, (8) sharing responsibility for conducting/designing research, (9) minimizing disruption to the community, (10) informing the community about the study and research in general, (11) identifying, mobilizing, and developing relevant community assets and capacity, (12) ensuring adequate opportunity and respect for dissenting opinions, (13) securing permission or authorization from the community, (14) attempting to ensure the research will not contribute to or reinforce disadvantage/stigma, and (15) transforming the relationship with the community in a way that is valuable for its own sake.

2 Even sources which could, but do not, recommend transformational engagement as a uniform practice sometimes include similar language. Take the following language from the UNAIDS Ethical Considerations in Biomedical HIV Prevention Trials: “The nature of community involvement should be one of continuous mutual education and respect, partnership, and consensus‐building regarding all aspects of the testing of potential biomedical HIV prevention products… All participating parties should define the nature of this ongoing relationship” [18, p. 22]. Taken literally, this seems to imply that engagement ought to include some process for obtaining the consent from the community; after all, proceeding without community consent would not involve building consensus and seems inconsistent with the ongoing relationship being mutually defined. But this implication is not codified in any concrete guidance point.

3 Note that while she characterizes this approach as consistent with the spirit of the federal regulations, she herself endorses a different “reasonable risk” standard, drawn from tort law; she takes it to be preferable for reasons related to those we are about to explore.

4 Under the US Code of Federal Regulations, it is possible to obtain waivers of consent for minimal risk research where requiring consent would render the research infeasible. However, arguing that obtaining consent is infeasible specifically because potential participants would be likely to say “no,” because even though the risks are minimal they would still prefer not to undergo them, arguably falls afoul of the requirement that waivers must not adversely affect the rights or welfare of participants. By contrast, traditionally acceptable reasons to treat minimal risks research as infeasible without a waiver of consent include, for example, that recontacting individuals on the basis of decades‐old medical records is impossible within a reasonable timeframe or budget.

5 What, instead, does explain the right to decline research participation, if not a general right against risk imposition? Here is one potential answer: participants have natural rights against certain forms of bodily intrusion and privacy invasion which are typically required by research, as well as some additional conventional protections on top of that established through law and regulation, and together these empower them to reject all or almost all research participation.

6 Macer, despite the earlier quoted statement, ultimately suggests the need to develop a community‐level consent process that falls short of requiring unanimity for reasons similar to those just canvassed: “There will always be some people who are against any proposition, no matter how much others value it… a procedure that is neither paternalistic nor paralytic needs to be developed” [23, p. 656].

7 This quote is subsequent to an explicit rejection of an individualist approach. Note that Neuhaus also embraces a broad conception of being affected by a trial that includes, for example, that trial bearing on non‐health‐related values regarding the relationship between humanity and nature [24, p. 33–34]. But if value (dis)alignment with the research counts as a way of being affected, this will presumably also apply to clinical trials. Plausibly this requirement should therefore hold more broadly if it holds at all.

8 Relatedly, some have argued that community consent is inapposite unless the community in question has a political structure that empowers individuals to give consent on behalf of the collective and have pointed out that many for example, disease groups or ethnic minorities do not have that kind of structure [25, 26]. This is correct in the intended sense: a disease group does not have that structure all to its own. But it does have that structure in another sense; it has the political structure of being part of a national state which empowers national officials to give consent on behalf of the whole.

9 Relatedly, see Heath on some of the limits of participatory methods in securing accountability in administrative decisions [28, p. 72–9].

10 While the arguments to follow do not require endorsing any particular threshold, it is worth noting Picchio's argument that the relevant threshold for a related question—the authorization of policy experiments, specifically—is whether a state counts as a minimally egalitarian democracy [29].

11 As the WHO advises: “[p]eople from key populations can also transmit HIV to other populations—for example, sex workers' clients and the sexual partners of people who inject drugs. Thus, infections in people from key populations can have a multiplier effect… Projections suggest that the impacts of improved service coverage of key populations could range from averting a considerable number of new infections in countries with generalized epidemics to stabilizing or even turning around growing incidence rates in countries with concentrated epidemics” [31, p. 8–9].

12 Consider the GPP guidelines' definition [8, p. 14–15]: “Stakeholders are individuals, groups, organizations, government bodies, or any other individuals or collections of individuals who can influence or are affected by the conduct or outcome of a biomedical HIV prevention trial. In this guidance document, the term ‘stakeholders’ is all‐encompassing. It describes any individual or collection of individuals who have a stake in a biomedical HIV prevention trial.” The guidelines also define “community stakeholders,” specifically, as “individuals and groups that are ultimately representing the interests of people who would be recruited to or participate in a trial, and others locally effected by a trial. Examples of ‘community stakeholders' are the population to be recruited, trial participants, people living in the area where the research is conducted, people living with HIV in the area, local HIV‐positive groups or networks, people in the area who are affected by the HIV epidemic, local non‐governmental organizations, community groups, and community‐based organizations.” While community stakeholders are a narrower class—one that explicitly excludes funders, sponsors, trial implementers, and governments—it is still extremely broad, and arguably literally everyone will fall under the category of being ‘affected by the HIV epidemic,’ given that it has universal social, commercial, and health effects.

13 The following account is based on the two cited reports, which are comprehensive retrospectives that were compiled by the Global Campaign for Microbicides and informed by research and interviews with dozens of individuals representing different perspectives from across the involved conflicts. The Global Campaign for Microbicides closed in 2012 and no longer hosts these pdfs, hence the citation to internet archive links. Both reports remain cited in the GPP guidelines [8].
